# Physiological responses during simulated 16 km recumbent handcycling time trial and determinants of performance in trained handcyclists

**DOI:** 10.1007/s00421-020-04390-w

**Published:** 2020-05-20

**Authors:** Benjamin Stone, Barry S. Mason, Ben T. Stephenson, Vicky L. Goosey-Tolfrey

**Affiliations:** 1 Peter Harrison Centre for Disability Sport, School of Sport, Exercise and Health Sciences, Loughborough University, NCSEM 1.26, Loughborough University Campus, Loughborough, LE11 3TU UK; 2 English Institute of Sport, Performance Centre, Loughborough University, Loughborough, UK

**Keywords:** Endurance performance, Paralympic, Lactate threshold, Disability sport, Spinal cord injury

## Abstract

**Purpose:**

To characterise the physiological profiles of trained handcyclists, during recumbent handcycling, to describe the physiological responses during a 16 km time trial (TT) and to identify the determinants of this TT performance.

**Methods:**

Eleven male handcyclists performed a sub-maximal and maximal incremental exercise test in their recumbent handbike, attached to a Cyclus II ergometer. A physiological profile, including peak aerobic power output (PO_Peak_), peak rate of oxygen uptake ($\dot{V}$O_2Peak_), aerobic lactate threshold (AeLT) and PO at 4 mmol L^−1^ (PO_4_), were determined. Participants also completed a 16 km simulated TT using the same experimental set-up. Determinants of TT performance were identified using stepwise multiple linear regression analysis.

**Results:**

Mean values of PO_Peak_ = 252 ± 9 W, $\dot{V}$O_2Peak_ = 3.30 ± 0.36 L min^−1^ (47.0 ± 6.8 mL kg^−1^ min^−1^), AeLT = 87 ± 13 W and PO_4_ = 154 ± 14 W were recorded. The TT was completed in 29:21 ± 0:59 min:s at an intensity equivalent to 69 ± 4% PO_Peak_ and 87 ± 5% $\dot{V}$O_2Peak_. PO_Peak_ (*r* = − 0.77, *P* = 0.006), PO_4_ (*r* = − 0.77, *P* = 0.006) and AeLT (*r* = − 0.68, *P* = 0.022) were significantly correlated with TT performance. PO_4_ and PO_Peak_ were identified as the best predictors of TT performance (*r* = 0.89, *P* < 0.001).

**Conclusion:**

PO_Peak_, PO_4_ and AeLT are important physiological TT performance determinants in trained handcyclists, differentiating between superior and inferior performance, whereas $\dot{V}$O_2peak_ was not. The TT took place at an intensity corresponding to 69% PO_Peak_ and 87% $\dot{V}$O_2peak_.

## Introduction

Recumbent handcycling is an endurance sport for athletes with lower limb impairments, such as spinal cord injuries, lower limb amputations and congenital conditions (Abel et al. 2006). Since 2004 (Athens Paralympic Games), handcycling has been an integral part of the paracycling road programme, with 65 handcyclists contesting 13 events at the most recent 2016 Paralympic Games in Rio (Paralympics.org 2016). At national and international events (e.g. British Championships, Paralympic Games), handcyclists compete in individual time trials (TT) (10–30 km, lasting 20–40 min) and/or road races (40–80 km, lasting 60–150 min) with many handcycling events being scheduled on consecutive days (Zeller et al. 2017). To date, most studies examining physiological responses during handcycling have gathered and described heart rate (HR), capillary blood lactate concentration (BLa), power output (PO) or rate of oxygen uptake ($\dot{V}$O_2_) during laboratory-based exercise tests (Janssen et al. 2001; Abel et al. 2010; de Groot et al. 2014; Kouwijzer et al. 2018b; Quittmann et al. 2018). These studies have been limited to recreational or touring bike configurations which are disparate to that of elite handcyclists. It is only recently that recumbent handcycling studies have been conducted during competition (West et al. 2015) or protocols replicating recumbent handcycling or competitive race intensities (Fischer et al. 2015, 2020; Graham-Paulson et al. 2016; Stangier et al. 2019; Stone et al. 2019b).

Physiological variables, such as peak rate of oxygen uptake ($\dot{V}$O_2Peak_) and ventilatory thresholds are the best predictors of handcycling TT performance. Moreover, peak power output achieved during incremental tests to exhaustion (PO_Peak_) has been related to performance (Janssen et al. 2001; Lovell et al. 2012; de Groot et al. 2014; Fischer et al. 2015). However, large variance in PO_Peak_ (129–247 W) and $\dot{V}$O_2Peak_ (2.0–3.45 L min^−1^ equivalent to 26.5–42.3 mL kg^−1^ min^−1^) have been reported in the literature (Janssen et al. 2001; Abel et al. 2006; Lovell et al. 2012; Fischer et al. 2015; Graham-Paulson et al. 2018; Kouwijzer et al. 2018a; Stangier et al. 2019; Stone et al. 2019a). Confounding factors, such as training status, athlete classification and the number of years involved in the sport and indeed the protocols adopted (e.g. ramp vs. step incremental test), all contribute to these reported physiological profiles (Miller et al. 2004; Goosey-Tolfrey et al. 2008; van Drongelen et al. 2009; Lovell et al. 2012). Moreover, since the experimental designs of these studies varied considerably, using attachable-units, touring handbikes or bespoke ergometers, this makes the findings difficult to transfer to the recumbent handbikes used in the present day. These handbikes are considerably lighter, but more importantly, are bespoke to the individual, with respect to crank position, crank width and backrest inclination (Stone et al. 2018). Therefore, the purpose of this study was to characterise the physiological profiles of trained handcyclists, during recumbent handcycling and to identify which physiological variables are related to 16 km TT performance.

## Methods

### Participants

Eleven male recumbent handcyclists (age 38 ± 10 years; body mass 72 ± 9 kg; classification 5 H3 and 6 H4; injury description 4 spinal cord lesions complete (4th–11th thoracic vertebra), 3 spinal cord lesions incomplete (8th–9th thoracic vertebra), 3 lower limb amputees and 1 diplegic cerebral palsy) volunteered to participate. All participants had competed in handcycling or paratriathlon, at a national or international level (handcycling experience 5.4 ± 5.6 years; training duration 5 ± 4 handcycling sessions totalling 7 ± 3 h week^−1^ with a self-reported distance of 178 ± 93 km week^−1^ and 2 ± 1 gym sessions totalling 3 ± 2 h week^−1^). The University’s local ethics committee approved the study. Before participation, all participants provided their written, informed consent.

### Experimental protocol

**Fig. 1:**
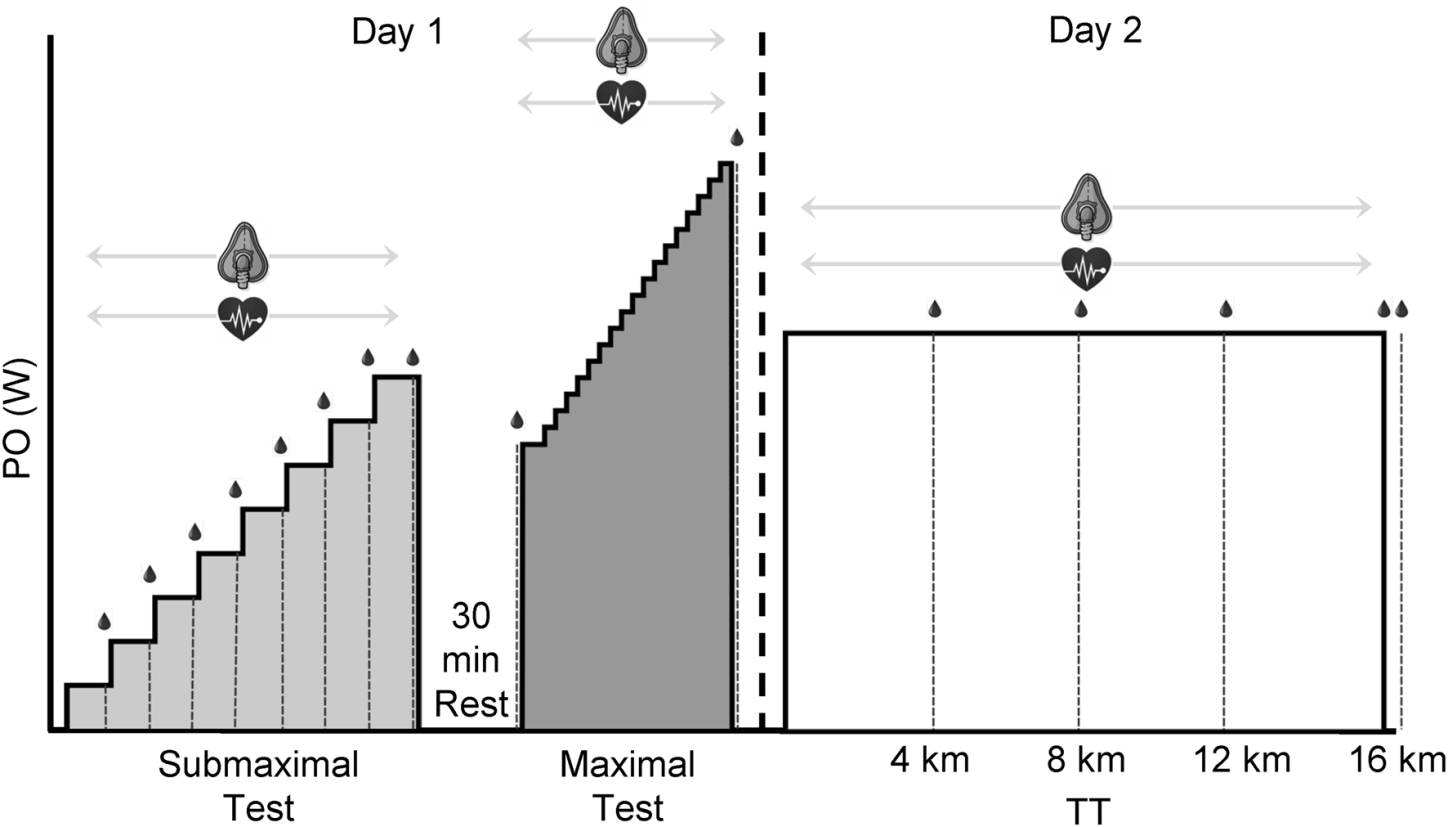
Experimental protocol for the submaximal test, maximal test and TT with details of the collection of BLa, HR and $\dot{V}$O_2_

Participants refrained from exercise in the 24 h preceding the testing. The experiment protocol was performed on two consecutive days (Fig. 1). On the first day, participants completed a sub-maximal exercise test, to determine—the aerobic threshold (AeLT), power output (PO) at 4 mmol L^−1^ (PO_4_), and maximal incremental exercise test, to assess PO_Peak_, $\dot{V}$O_2Peak_, and peak heart rate (HR_Peak_). Ventilatory thresholds were not determined as there has been mixed results to their suitability in this sample (Kouwijzer et al. 2019; Leicht et al. 2014). Both tests were conducted in the participants’ bespoke recumbent handbike (5 ‘Carbonbike’, 4 ‘Top End’ and 2 ‘Schmicking’), which were attached to a Cyclus II ergometer (RBM electronic automation GmbH, Leipzig, Germany). Following a 10-min warm-up at a self-selected cadence and intensity, the sub-maximal test commenced with an initial load of 20 W, increasing by 20 W every 4 min until BLa exceeded 4.0 mmol L^−1^ when the test was terminated. BLa was determined from 20 μL earlobe capillary blood samples, collected in the last minute of each stage and analysed using a Biosen C-Line (EFK Diagnostics GmbH, Germany). Breath-by-breath gas analysis (Cortex Metalyzer 3B, Cortex, Leipzig, Germany)—for calculation of $\dot{V}$O_2_, carbon dioxide production and respiratory exchange ratio (RER)—HR (Polar RS400, Kempele, Finland) and rating of perceived exertion (RPE; Borg 1998), were also collected in the last minute of each stage. AeLT was calculated using the log–log transformation method (Beaver et al. 1985). Moreover, this test enabled the identification of the PO corresponding to a fixed BLa of 4 mmol L^−1^ (PO_4_), by using linear interpolation methods as used in the handcycling literature (Stangier et al. 2019; Zeller et al. 2017). Following 30 min’ passive rest, participants performed a maximal test to exhaustion. The starting PO was equivalent to their AeLT and was maintained for 2 min. PO was then increased by 5 W every 15 s until the athlete reached volitional exhaustion (failure to maintain cadence ≥ 50 rpm and an overall RPE ≥ 19) (Graham-Paulson et al. 2016). Verbal encouragement was provided during this test. Breath-by-breath gas analysis and HR were recorded continuously throughout the maximal test and BLa reported 2 min post-completion of the maximal test. The ergometer was set in a power control mode, ensuring the pre-set PO was controlled independently of cadence or gear selection. Cadence was self-selected in all trials as reported elsewhere (Graham-Paulson et al. 2016).

On the second day, participants completed a 16-km TT in the shortest time possible. Participants selected the gear ratio to commence the TT, which could then be changed virtually by the investigators throughout the time trial, as instructed by participants. No motivation was provided during the TT and the feedback provided was PO, cadence and cumulative distance displayed on the ergometer, to maximise ecological validity. Breath-by-breath gas analysis and HR were recorded continuously throughout the TT and BLa collected every 4 km.

### Data and statistical analysis

In the sub-maximal exercise test $\dot{V}$O_2_, HR, RER and cadence were averaged across the last minute of each stage. Mechanical efficiency (ME%), calculated as the ratio of external work to energy expended (Powers et al. 1984), in 1 min of exercise, was determined at AeLT and PO_4_ (RER < 1.00 for all participants). Energy expenditure was calculated from the product of $\dot{V}$O_2_ and RER and the standard conversion table (Péronnet and Massicotte 1991). During the maximal exercise test, the highest 30-s rolling average of $\dot{V}$O_2_ and HR were used to calculate $\dot{V}$O_2Peak_ and HR_Peak_. A 15-s rolling average was used to calculate PO_Peak_. In the TT, $\dot{V}$O_2_, HR, RER, cadence, PO, speed and ME% were averaged every km and calculated relative to $\dot{V}$O_2Peak_, HR_Peak_ and PO_Peak_, where applicable.

Shapiro–Wilks tests were used to determine the distribution of the data. Repeated measures analysis of variance was used to determine differences in $\dot{V}$O_2_, HR, PO and cadence across each km of the TT. Sphericity was assessed using the Mauchly’s test of sphericity. If the data were aspherical, a correction, using the Greenhouse–Geisser epsilon, was applied to the calculated *P* value (Girden 1992). If a significant difference was identified, post hoc paired *t* tests, with Bonferroni corrections, were applied to determine the differences in cadence or PO.

Pearson’s product-moment correlation was used to analyse the relationship between variables from the sub-maximal (AeLT, ME% at AeLT, $\dot{V}$O_2_ at AeLT, PO_4_, ME% at PO_4_ and $\dot{V}$O_2_ at PO_4_) and maximal test [$\dot{V}$O_2Peak_ (absolute and relative), HR_Peak_ and PO_Peak_] to TT performance (IBM SPSS Statistics 24, Chicago, IL). Variables that were significantly correlated with 16 km TT performance were inputted into a stepwise multiple linear regression to establish the most important determinants of 16 km TT performance. Durbin–Watson test was used to indicate the independence of variables entered into the regression. Tolerance and variance inflation factors were also used to assess multicollinearity within the data.

## Results

### Outcomes of the exercise tests and TT

**Fig. 2:**
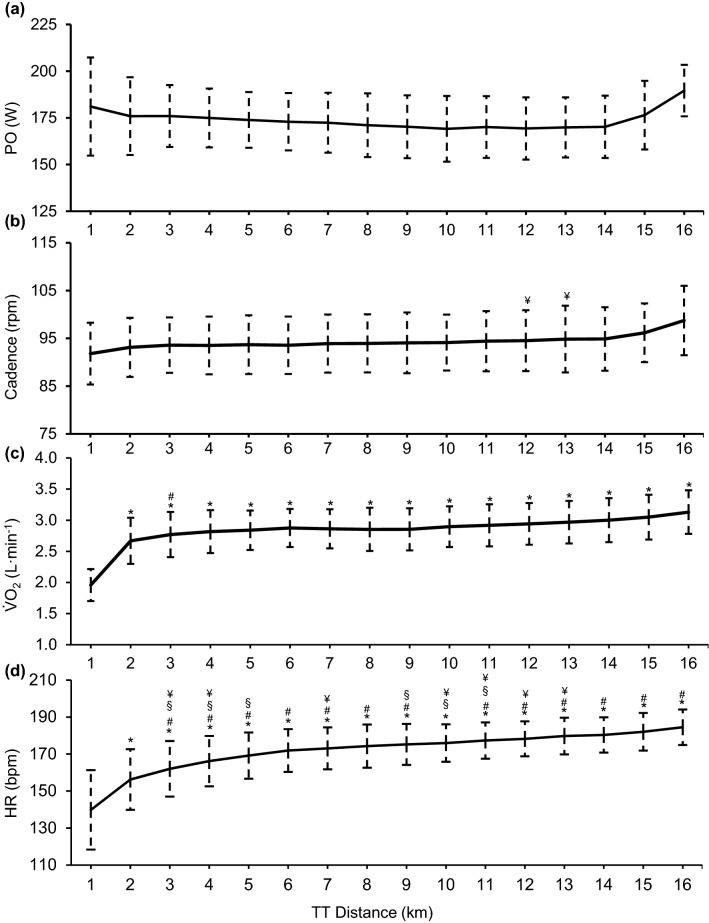
PO (**a**), cadence (**b**), $\dot{V}$O_2_ (**c**) and HR (**d**) responses of trained recumbent handcyclists throughout a 16-km TT (values: mean ± SD). *Significant difference to 1st km (*P* < 0.05), ^#^Significant difference to 2nd km (*P* < 0.05), ^§^Significant difference to 15th km (*P* < 0.05), ^¥^Significant difference to 16th km (*P* < 0.05)

Data from the sub-maximal exercise test, maximal exercise test and 16 km TT are summarised in Table 1. No significant differences in PO were identified throughout the TT (Fig. 2a). The 4-km sector split times ranged from 7:24 ± 0.14 min:s (4–8 km) to 7:17 ± 0.14 min:s (12–16 km), further indicating that, as a group, the handcyclists maintained a relatively constant pace throughout the TT. Cadence increased throughout the TT, and during the 16th km was on average 4 rpm greater than the 12th and 13th km (*P* < 0.05) (Fig. 2b). $\dot{V}$O_2_ increased significantly during the first 2 km of the TT (*P* < 0.05) before plateauing during the remainder of the TT (Fig. 2c). Similarly, HR significantly increased during the first 3 km of the TT (*P* < 0.05) before plateauing between the 3rd km and 14th km and significantly increasing in the final 2 km (*P* < 0.001) (Fig. 2d). BLa significantly increased throughout the TT [4 km: 8.0 ± 2.3 mmol L^−1^; 8 km: 10.0 ± 2.6 mmol L^−1^; 12 km: 11.2 ± 2.7 mmol L^−1^; 16 km: 13.1 ± 2.7 mmol L^−1^ (*P* < 0.001)], while ME% was found to significantly decrease [0–4 km: 19.3 ± 1.2%; 4–8 km: 16.8 ± 1.2%; 8–12 km: 16.3 ± 1.3%; 12–16 km: 16.3 ± 1.5% (*P* < 0.001)]. The average intensity of the TT’s was equivalent to 69 ± 4% of PO_Peak_ and 87 ± 5% of $\dot{V}$O_2Peak_ (Table 1).

**Table 1:** Physiological responses from the submaximal and maximal recumbent handcycling tests and the average response from the whole 16 km handcycling TT (*n* = 11)

Parameter	Mean ± SD	Min	Max
Submaximal test			
AeLT (W)	87 ± 13	70	108
$\dot{V}$O_2_ at AeLT (L min^−1^)	1.49 ± 0.12	1.29	1.63
ME% at AeLT (%)	16.9 ± 1.6	14.5	19.8
PO_4_ (W)	154 ± 14	128	173
$\dot{V}$O_2_ at PO_4_ (L min^−1^)	2.43 ± 0.40	1.92	3.27
ME% at PO_4_ (%)	17.9 ± 1.7	14.3	19.6
Maximal test			
$\dot{V}$O_2Peak_ (L min^−1^)	3.30 ± 0.36	2.75	4.02
$\dot{V}$O_2Peak_ (mL kg^−1^ min^−1^)	46.98 ± 6.80	36.31	57.64
PO_Peak_ (W)	252 ± 19	229	282
BLa (mmol L^−1^)	10.90 ± 2.51	7.48	14.28
HR_Peak_ (bpm)	188 ± 11	169	208
RER_Peak_	1.15 ± 0.07	1.06	1.26
16 km TT			
Time (min:s)	29:20.7 ± 00:58.8	27:58.0	30:33.1
Velocity (km h^−1^)	32.7 ± 1.1	31.4	34.3
PO (W)	174 ± 15	152	190
Cadence (rpm)	94.3 ± 6.0	84.3	102.0
HR (bpm)	171 ± 12	154	194
$\dot{V}$O_2peak_ (L min^−1^)	2.84 ± 0.31	2.37	3.26
BLa (mmol L^−1^)	10.5 ± 2.5	7.3	15.0
%$\dot{V}$O_2Peak_	87.07 ± 5.03	79.65	92.66
%HR_Peak_	92.34 ± 3.23	86.66	97.52
%PO_Peak_	68.83 ± 3.78	64.51	74.93
ME (%)	17.1 ± 1.3	14.7	18.5

### Determinants of TT performance

The highest correlations were observed between PO_Peak_ (*r* = − 0.77, *P* = 0.006), PO_4_ (*r* = − 0.77, *P* = 0.006), AeLT (*r* = − 0.68, *P* = 0.022) and TT performance (Table 2). Moderate to low correlations were observed for all other physiological parameters (Table 2). The multiple linear regression indicated that PO_4_ was the strongest predictor accounting for 59.3% of the variance in 16 km TT performance (*P* = 0.006). The addition of PO_Peak_ (19.0% of overall variance) to PO_4_ provided a stronger prediction model, accounting for 78.3% of the variance in performance (*P* = 0.002). A Durbin–Watson value of 1.501 indicated that variables within this model were sufficiently independent and not autocorrelated. Tolerance values of 0.741 and a variance inflation factor of 1.350 further indicate that multicollinearity was not an issue for this regression model.

**Table 2:** Correlations between 16 km TT and physiological parameters determined in the submaximal and maximal exercise tests (*n* = 11)

Parameter	Correlation coefficient (*R*)	*P*
Sub-maximal test		
AeLT (W)	− 0.68*	0.022
$\dot{V}$O_2_ at AeLT (L min^−1^)	− 0.43	0.191
ME at AeLT (%)	− 0.38	0.256
PO_4_ (W)	− 0.77*	0.006
$\dot{V}$O_2_ at PO_4_ (L min^−1^)	− 0.24	0.472
ME at PO_4_ (%)	− 0.30	0.370
Maximal test		
PO_Peak_ (W)	− 0.77*	0.006
HR_Peak_ (bpm)	− 0.53	0.095
$\dot{V}$O_2Peak_ (L min^−1^)	− 0.06	0.868
$\dot{V}$O_2Peak_ (mL kg^−1^ min^−1^)	− 0.21	0.539

* Correlation *P* < 0.05

## Discussion

The current study investigated the physiological responses during a 16-km TT, in a population of national and international recumbent handcyclists. Extending the recent work of Stangier et al. (2019), this study focused on recumbent trained handcyclists yet was designed to measure the sub-maximal exercise, maximal exercise and simulated TT performance in their own bespoke recumbent handbikes. A key finding was that better 16 km TT performances were achieved by handcyclists with a higher PO_Peak_, PO_4_ and AeLT. Conversely, $\dot{V}$O_2Peak_, both absolute and relative, were not correlated with TT performance.

The results from the maximal exercise test revealed that the handcyclists in the current study had higher average and maximal $\dot{V}$O_2Peak_ and PO_Peak_ values than previously reported (Lovell et al. 2012; Fischer et al. 2015; Graham-Paulson et al. 2018; Stangier et al. 2019). However, the participants in the current study, with $\dot{V}$O_2Peak_ and PO_Peak_ values of 46.9 ± 6.8 mL kg^−1^ min^−1^ and 252 ± 19 W, respectively, were similarly trained (averaging ≥ 7 h week^−1^ and ≥ 175 km week^−1^) to the participants from previous studies (Lovell et al. 2012; Fischer et al. 2015; Stangier et al. 2019). Furthermore, the handcyclists that achieved the greatest $\dot{V}$O_2Peak_ (4.02 L min^−1^ and 57.6 mL kg min^−1^) and PO_Peak_ (281 W) values averaged 5 handcycling sessions a week totalling 8 h week^−1^ with a self-reported distance of 170 km week^−1^. Therefore, the higher physiological values observed in the current study are likely to be due to advances in endurance training regimes (Zeller et al. 2017), strength training (Nevin et al. 2018) and the use of bespoke recumbent handbikes in comparison to the modified ergometers used previously (Lovell et al. 2012; Fischer et al. 2015). Participants were likely to be more stable and comfortable in their handbike, which is perceived to be essential for the application of power in handcycling, potentially facilitating the increased $\dot{V}$O_2Peak_ and PO_Peak_.

The trained handcyclists completed the simulated 16 km TT at a similar intensity (87 ± 5% $\dot{V}$O_2Peak_) to a 20 and 22 km TT, reported by Graham-Paulson et al. (2018) and Fischer et al. (2015), respectively, yet with much higher end BLa (> 6 mmol L^−1^). During the TT participants maintained a constant PO, similar to Fischer et al. (2015), although ME% reduced from 19.3% in the first 4 km to 16.3% in the final 4 km. Based on this data, future constant work studies replicating handcycling TTs, should select a PO equivalent to ~ 70% PO_Peak_ or ~ 25% greater than PO_4_ (using methods similar to the current study). This high exercise intensity indicates the contribution of the anaerobic pathways to supply energy during a typical handcycling TT. Therefore, a high anaerobic capacity may play an important role in handcycling TT success, like able-bodied cycling (Støren et al. 2013), although this requires future investigation.

TT performance was significantly and highly correlated with PO_Peak_ (*r* = − 0.77, *P* = 0.006), which supports previous research (Janssen et al. 2001; Lovell et al. 2012; de Groot et al. 2014; Fischer et al. 2015). The present study found, for the first time, that recumbent handcycling 16 km TT performance was strongly correlated with AeLT (*r* = − 0.68, *P* = 0.022) and PO_4_ (*r* = − 0.77, *P* = 0.006). Contrary to the previous handcycling literature (Janssen et al. 2001; Lovell et al. 2012; de Groot et al. 2014; Fischer et al. 2015), absolute $\dot{V}$O_2Peak_ (*r* = − 0.06) and relative $\dot{V}$O_2Peak_ (*r* = − 0.21) were not correlated with TT performance. Therefore, these results suggest that, in highly trained handcyclists with a heterogenous injury description, PO_Peak_ and blood lactate (AeLT and PO_4_) are better predictors of TT performance than $\dot{V}$O_2Peak_. In able-bodied athletes, fractional utilisation of $\dot{V}$O_2Peak_, i.e. intensity at lactate threshold, has long been understood to better predict endurance performance in highly trained athletes whilst PO_peak_ has consistently been shown to predict cycling performance, regardless of task duration or test profile (Bentley et al. 2001; Joyner and Coyle 2008). This has also been shown to be true when assessing physiological correlates to cycling performance within a paratriathlon race (Stephenson et al. 2020). As such, this finding is not unique in endurance sport but is presently confirmed in elite handcyclists.

The current study extends previous work, which was limited to handcyclists with a spinal cord injury (Lovell et al. 2012; Fischer et al. 2015; West et al. 2015; Graham-Paulson et al. 2018), by including participants with lower limb impairments and cerebral palsy, which is more representative of athletes competing at the Paralympic Games. The increased recruitable muscle mass of athletes with non-paralysed lower limbs (e.g. amputation or cerebral palsy), results in greater rates of oxygen uptake relative to athletes with complete spinal lesions (Baumgart et al. 2018). For example, in amputee athletes this is due to the capability to use lower limb musculature to brace within the handbike for greater stability and power transfer. The increased available muscle mass and the potential loss of lower limbs makes variables such as absolute $\dot{V}$O_2Peak_, relative $\dot{V}$O_2Peak_ and power to weight ratio unrepresentative or misleading without scaling parameters (Goosey-Tolfrey et al. 2003). Therefore, in a population of handcyclists competing in the H3 and H4 classes [spinal lesions, lower limb amputations and cerebral palsy (UCI 2019)] variables such as PO_Peak_ and blood lactate (AeLT and PO_4_) are better indicators of TT performance than $\dot{V}$O_2Peak_. From this data, it may be recommended that testing for PO_Peak_ or PO_4_ is conducted with handcyclists to infer performance potential. Although assessing the latter requires specialist equipment for BLa measurement, PO_4_ is also commonly used for training intensity prescription.

## Limitations

An electronically braked ergometer was used to simulate the TT. The electronic braking meant that handcyclists had to pedal continually to apply power, which differs from a road TT. The corners and gradients throughout a road TT course would allow the handcyclists to recover for short periods (0–5 s). Furthermore, as the ergometer was fixed to the floor, the influence of aerodynamic drag, steering and braking were removed. Future research should aim to collect TT data on the road, in a competition if possible, and compare with laboratory-based findings. Additionally, recruiting participants from the H1, H2 and H5 classes, along with a greater number within H3 (*n* = 5) and H4 (*n* = 6) would allow a comparison between classes. Finally, the inter-individual variability of physiological parameters in the study’s cohort is acknowledged. Heterogeneity is inherent in Paralympic populations due to athletes’ spectra of impairments. Furthermore, variation in physiological parameters are likely largely mediated by training history and performance standard.

## Conclusions

The current study revealed that the best predictors of 16 km TT performance, in a population of trained recumbent handcyclists, were PO_4_ and PO_Peak_. It is suggested that PO_4_ and PO_Peak_ are used to infer performance level/potential rather than $\dot{V}$O_2Peak_ within a population of H3 and H4 recumbent handcyclists (spinal cord lesion vs. lower limb amputation vs. cerebral palsy). A protocol equivalent to 70% PO_Peak_ is recommended, in future studies, to replicate 16 km TT intensity.

## Abbreviations

AeLT: Aerobic threshold
PO_4_: Power output at 4 mmol L^−1^
BLa: Blood lactate concentrations
HR: Heart rate
$$\dot{V}$$O_2_: Oxygen uptake
ME%: Mechanical efficiency
PO: Power output
PO_Peak_: Peak aerobic power output
HR_Peak_: Peak heart rate
$\dot{V}$O_2Peak_: Peak rate of oxygen uptake
RPE: Rating of perceived exertion
RER: Respiratory exchange ratio
TT: Time trial
